# The macroevolutionary landscape of short-necked plesiosaurians

**DOI:** 10.1038/s41598-020-73413-5

**Published:** 2020-10-02

**Authors:** Valentin Fischer, Jamie A. MacLaren, Laura C. Soul, Rebecca F. Bennion, Patrick S. Druckenmiller, Roger B. J. Benson

**Affiliations:** 1 Evolution & Diversity Dynamics Lab, Université de Liège, 14 Allée du 6 Août, 4000 Liège, Belgium; 2 Department of Paleobiology, Smithsonian Institution, P.O. Box 37012, Washington, DC 20013-7012 USA; 3 OD Earth and History of Life, Institut Royal des Sciences Naturelles de Belgique, 29 Rue Vautier, 1000 Brussels, Belgium; 4 University of Alaska Museum and Department of Geosciences, University of Alaska Fairbanks, 1962 Yukon Drive, Fairbanks, AK 99775 USA; 5 Department of Earth Sciences, University of Oxford, South Parks road, Oxford, OX1 3AN UK

**Keywords:** Palaeontology, Evolutionary ecology, Palaeoecology, Biodiversity

## Abstract

Throughout their evolution, tetrapods have repeatedly colonised a series of ecological niches in marine ecosystems, producing textbook examples of convergent evolution. However, this evolutionary phenomenon has typically been assessed qualitatively and in broad-brush frameworks that imply simplistic macroevolutionary landscapes. We establish a protocol to visualize the density of trait space occupancy and thoroughly test for the existence of macroevolutionary landscapes. We apply this protocol to a new phenotypic dataset describing the morphology of short-necked plesiosaurians, a major component of the Mesozoic marine food webs (ca. 201 to 66 Mya). Plesiosaurians evolved this body plan multiple times during their 135-million-year history, making them an ideal test case for the existence of macroevolutionary landscapes. We find ample evidence for a bimodal craniodental macroevolutionary landscape separating latirostrines from longirostrine taxa, providing the first phylogenetically-explicit quantitative assessment of trophic diversity in extinct marine reptiles. This bimodal pattern was established as early as the Middle Jurassic and was maintained in evolutionary patterns of short-necked plesiosaurians until a Late Cretaceous (Turonian) collapse to a unimodal landscape comprising longirostrine forms with novel morphologies. This study highlights the potential of severe environmental perturbations to profoundly alter the macroevolutionary dynamics of animals occupying the top of food chains.

## Introduction

Amniotes are ’land vertebrates’, but have nevertheless undergone at least 69 independent evolutionary transitions from land into aquatic environments^1^. Sea-going (marine) amniotes are textbook examples of inter- and intraclade convergent evolution, with repeated acquisitions of short, hydrodynamic body plans^2–9^. The extinct but diverse, Mesozoic clade Plesiosauria provides a striking example of repeated convergence in marine amniotes in the form of transitions from a long-necked body plan to a large-headed, short-necked body plan, which occurred several times during their long evolutionary history^9–12^.

**Figure 1:**
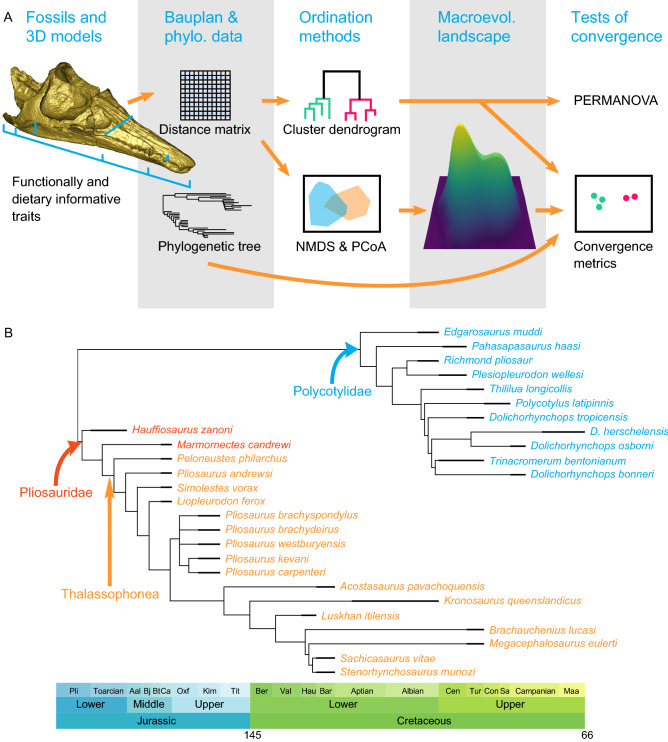
Protocol for reconstructing and testing a macroevolutionary landscape using phenotypic data. (**A**) Workflow, showing the skull is a three-dimensional model of the holotype of *Plesiopleurodon wellesi*, an occultonectian polycotylid from the early Late Cretaceous of the USA. (**B**) Strict consensus of the most parsimonious tree pruned to match the taxa used in our analyses.

However, these convergent morphologies are crudely generalised macroevolutionary pathways, inferred from a continuum of empirical variation. The extent to which they are supported by ecologically relevant traits, particularly craniodental characters, has not been determined thoroughly (but see^10, 13–17^ for recent attempts at quantification in Mesozoic marine reptiles, and upon which our study is based). To tackle this issue, we develop a protocol to analyse the patterns of morphospace occupation and convergence by using a series of quantitative tests and the density of phenotypes in a multivariate morphospace to approximate the macroevolutionary landscape (Fig. 1). We apply this protocol to analyse the evolution of short-necked plesiosaurians (Pliosauridae and Polycotylidae) over the entire Jurassic-Cretaceous interval (ca. 201 to 66 Mya). We find that the craniodental region of short-necked plesiosaurians evolved along a pervasive, bimodal landscape, separating large predators with robust craniodental morphologies from slender-snouted, longirostrine forms, which collapsed to a unimodal longirostrine landscape following profound environmental changes during the Late Cretaceous.

## Methods

### Data

**Figure 2:**
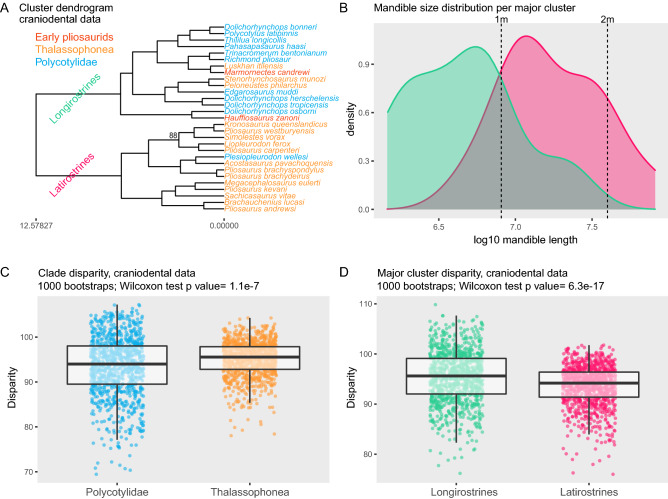
Two main craniodental morphotypes among short-necked plesiosaurians. (**A**) Cluster dendrogram. Values of node support (approximate unbiased p-value) are indicated when below 97%. (**B**) Distribution of mandible size per major morphotype; latirostrine plesiosaurians generally have (much) larger skulls than longirostrine forms. (**C**,**D**) comparisons of total disparity per clade (**C**) and per morphotype (**D**). The packages ggplot2 v3.3.1^21^, ggdendro v0.1-20^22^ , dendextend v.1.13.2.^23^, and gridextra v2.3.^24^ in the R v3.6.2 statistical environment^20^ (https://www.r-project.org) were used to produce this figure.

We gathered measurements for all pliosaurid and polycotylid species known to date with relevant cranial material (40 species; see Tables S1, S2 in Supplementary Information and Supplementary Files). We chose to score the moderately long-necked polycotylid *Thililua longicollis*, because of its fairly derived phylogenetic position and possession of craniodental features otherwise associated with short-necked plesiosaurians such as an elongated rostrum and carinated teeth^11^. The measurements were then used to create generate 11 morphofunctional traits (8 craniodental, 3 postcranial) describing the body plan of the sampled species (Fig. 1, Table S1). The apicobasal height of the tooth crown is the only absolute trait in the dataset (all others being ratios); this feature likely carries an important ecological signal, having a clear dietary relevance in modern marine amniotes (cetaceans)^18^. Mandible lengths were measured and used to scale datapoints in our figures (log-mandible length), and their distribution was visualised in Fig. 2. Measurements were obtained via several sources: by hand (either measured on the specimens by a digital calliper to the nearest 0.1 mm, or on high-resolution laser scans, see below), from the literature, from colleagues, and from high-resolution photographs (see^11, 19^ for previous versions of this dataset). We used a Creaform HandySCAN 300 laser surface scanner to create high-precision three-dimensional models of nine important specimens (resolution from 0.3 to 0.5 mm; see Table S2 for details; all the 3D models are deposited in Morphosource: http://www.morphosource.org/Detail/ProjectDetail/Show/project_id/1018). The original dataset has 42.05% missing entries and 39.37% when restricted to craniodental features, because of non-preservation of some elements and because of diagenetic compression. The reason why we report these data (see Supplementary Files) is to allow maximum dissemination of all the data we gathered, even if some taxa do not meet the standards for this study. Unless stated otherwise, all the results we report have been obtained on the craniodental dataset. All analyses were conducted in the R statistical environment^20^. We applied a 45% completeness threshold to each species to avoid distortions of the inferred morphospaces due to non-comparability issues resulting from highly incomplete specimens; eleven species did not pass the threshold for the craniodental dataset (Table S1); exclusion of these species reduced the amount of missing entries to 29.1% for the whole dataset, and to 23.27% for craniodental features. This pruned craniodental dataset includes 11 polycotylids, two non-thalassophonean pliosaurids, and 16 thalassophonean pliosaurids.

We obtained phylogenetic trees by analysing an updated version of the morphological data matrix of^11^ (originating from Benson and Druckenmiller^25^; see Supplementary Informationn for the details of the modifications). We analysed the dataset in TNT v1.5^26^ using the parsimony ratchet (ratchet: 200 iterations, drift and fuse activated, with 10 iterations each), within an implied weighting framework at k = 3. The maximum number of trees was set to 200,000; we used the tree branch swapping algorithm on the trees recovered by the parsimony ratchet to fully explore these shortest-trees islands. This resulted in 42,000 most parsimonious trees with a length of 143.03083. The most parsimonious trees (provided as a Supplementary File) differ in minor details of branch length, or that fall outside the taxa of interest. When pruned to match the taxa that passed the completeness threshold, all most parsimonious trees form a very well resolved strict consensus with a single polytomy involving the species of the genus *Pliosaurus* (Fig. 1). These minor differences did not result in noticeable differences in the results of downstream analyses. Accordingly, we present our results based on analyses performed on a single, randomly selected pruned tree, time-scaled using the ‘equal’ and ‘mbl = 3’ methods in the paleotree v.3.3.0 package^27^ (Fig. 1), using a set of taxon biostratigraphic ranges (provided as a Supplementary File).

### Ordination methods

**Figure 3:**
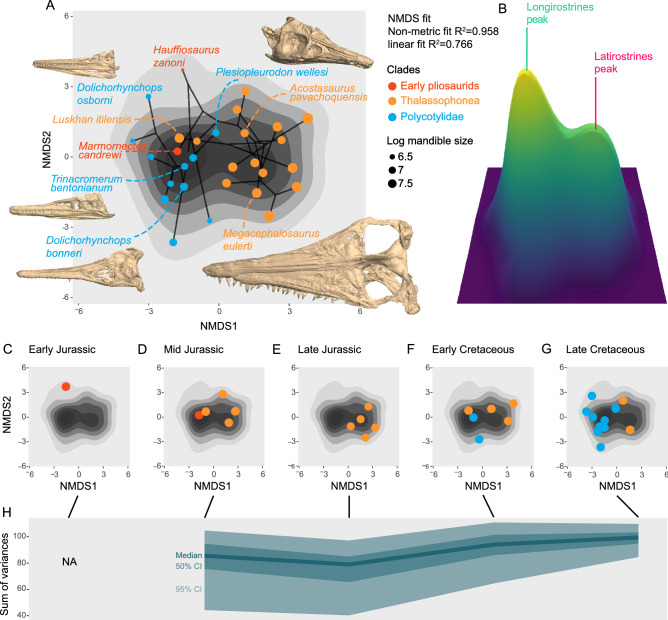
Craniodental morphospace occupation, disparity, and macroevolutionary landscape of short-necked plesiosaurians. (**A**) Phylomorphospace superimposed on the macroevolutionary landscape (NMDS). The 3D models are taxa from the Late Cretaceous of the Western Interior Seaway, USA and can be downloaded on Morphosource (http://www.morphosource.org/Detail/ProjectDetail/Show/project_id/1018). (**B**) Macroevolutionary landscape in oblique view (NMDS). (**C**–**G**) Morphospace (NMDS) occupation through time, superimposed on the macroevolutionary landscape (grey shades). (**H**) Temporal evolution of craniodental disparity (calculated as a sum of variance per bin), showing low values during the Late Jurassic and increasing values across the Jurassic-Cretaceous boundary and during the Cretaceous. The packages ggplot2 v3.3.1^21^, ggrepel v0.8.1^33^, gridextra v2.3.^24^ and plotly v4.9.1^34^ in the R v3.6.2 statistical environment^20^ (https://www.r-project.org) and Meshlab v.2020.7 (https://www.meshlab.net) were used to produce parts of this figure.

We z-transformed each morphological variable to have a mean of zero and a variance of 1, then computed a Euclidean distance matrix. We submitted this distance matrix to ordination methods and tests:

(i) We computed a cluster dendrogram (using a Ward clustering criterion “ward.D2” argument in hclust function; see Figs. 2, S2). We used the results from the cluster dendrogram to visualise the groups, noting that two main groups particularly stand out based on their ecomorphological traits (Fig. 2): ‘longirostrines’ and a group of robust predators for which we use the term ‘latirostrines’ (see explanation below). We used the psych v.1.8.12^28^ package to assess the intercorrelation of each body plan features (Fig. S1).
(ii) We assessed the statistical support of clusters using the ‘Approximately Unbiased P-value’ method of the pvclust v2.0 package^29^. This method employs multiscaled bootstrapping: instead of simply bootstrapping the dataset, it creates multiple datasets that can be smaller, equal to, and larger than the original dataset. This method is less biased than traditional bootstrapping^29^, and appears ideal for datasets that contain missing data^7^. We ran it from 0.5 times to 10 times the size of the original dataset, with 0.5 increments and 1000 bootstraps per increment.
(iii) We tested the strength of the latirostrines–longirostrines dichotomy using a permutational (non-parametric) multivariate analysis of variance (PERMANOVA^30^, using the vegan v.2.5-4 package^31^), which uses the distance matrix as the dependent variable. The independent variable was obtained directly from the cluster using the cutree function; 1000 permutations were employed. We also evaluated the significance of other groupings by applying PERMANOVA on successively smaller clusters, going from a k value of the cutree function of two (i.e. isolating the two main groups in the cluster dendrogram) to 10 (i.e. isolating the ten main groups in the cluster dendrogram) (Table S3). We analysed the correlation between the phenotypic distance and the phylogenetic position, and between the phenotypic distance and the geological age by computing Mantel tests (1000 permutations using the mantel function of the vegan v.2.5-6 package^31^) and by creating tanglegrams (dendextend v1.13.2 package^23^) (Fig. S1).
(iv) We also computed a principal coordinate analysis (PCoA; using Cailliez correction for negative eigenvalues using the ape v5.3 package^32^) and a non-metric multidimensional scaling (NMDS, dimensions = 2, using the vegan v.2.5-4 package^31^) (see Figs. 3, S2–S4), producing multivariate morphospaces.

### Macroevolutionary landscape

We computed the density of taxa in a two-dimensional morphospace (on both the NMDS and on the first two PCoA axes) using the kernel 2D density estimator of the MASS package v. 7.3-51.1^35^. This creates a third dimension that can be used to generate a three-dimensional landscape whose peaks thus represent commonly recurring phenotypes. The peaks in this visualisation are empirical, and do not necessarily correspond to optimal fitness (as in theoretical adaptive landscapes^36, 37^); however, we postulate that commonly recurring phenotypes approximate efficient skeletal architectures. It provides a hypothesised macroevolutionary landscape that can be statistically tested using other methods (described below). Two requirements were satisfied prior to computation of density to generate the landscape visualisation and a third to validate it (see also Fig. 1):

(i) Use of morphological data that has clear biomechanical and dietary implications, and that is independent of the cladistic characters used to generate the phylogenetic topology.
(ii) A clear, statistically significant separation of clusters, suggesting that a pervasive divide between groups exists in the dataset that potentially resulted from non-random patterns of phenotypic evolution along lineages, thereby reflecting an underlying macroevolutionary landscape.

We then tested whether this visualisation reflects an underlying macroevolutionary landscape that has structured patterns of intraclade convergent evolution:

(iii) Evidence of interclade convergence, suggesting that the distribution of species in morphospace does not simply reflect the occurrence of phylogenetically-distinct groups e.g.^38^ and can be statistically attributed to repeated instances of convergent evolution (see “Tests of convergent evolution” section).

The representation of density changes with the bandwidth used to calculate it; we found that a bandwidth equalling the maximal value on each axis yields the optimal visualisation, only slightly better than the default bandwidth of the MASS package v. 7.3-51.1^35^. The packages ggplot2 v.3.1.0^21^ and plotly v.4.8.0^34^ were used to visualize the resulting macroevolutionary landscapes (Figs. 3, S2). The exact position of the local maxima in 15 × 15 cell neighbourhood were computed using the raster package v3.0-7^39^. Finally, we also modified the ggphylomorphospace function of Barr^40^ to add a kernel 2D density estimator, so that phylogenies can be superimposed on the density-based empirical adaptive landscape in a ggplot framework (Figs. 3, S2; the script is provided as a Supplementary File).

### Tests of convergent evolution

Ordination methods and our visualisation described above suggested the possibility of interclade convergence, notably involving *Marmornectes candrewi* (an early pliosaurid^41^), *Luskhan itilensis* (a brachauchenine pliosaurid^19^), and *Trinacromeum bentonianum* (a polycotyline polycotylid^42^) among the longirostrine cluster, and *Acostasaurus pavachoquensis* (a brachauchenine pliosaurid^43^) and *Plesiopleurodon wellesi* (an occultonectian polycotylid^11^) among the latirostrine cluster. We tested the strength and significance of underlying patterns of convergent evolution based on exemplar cases (*Luskhan itilensis* + *Marmornectes candrewi* + *Trinacromerum bentonianum* for longirostrines; *Plesiopleurodon wellesi* + *Acostasaurus pavachoquensis* for latirostrines) using the C1, C2, C3, and C4 metrics of Stayton^44^. These test the strength of the convergence process in reducing the dissimilarity between lineages, considering the morphology of their ancestors. The C1 and C2 metrics compare the phenotypic distance of putatively convergent taxa (tips) to the pair of ancestors (nodes) showing the maximum phenotypic dissimilarity, while the C3 and C4 metrics also incorporate the total amount of phenotypic evolution from ancestors to descendants^44^. The significance of these metrics is assessed by simulating character evolution under Brownian motion 1000 times, using the convevol package v.1.3^45^. We applied Stayton’s metrics on a randomly selected most parsimonious tree, using the first two, the first five, and all axes of the PCoA as in Button and Zanno^46^. Contrary to Button and Zanno^46^, the number of axes used to analyse convergence in a multivariate space does not need to be limited for the C1 to C4 metrics (this only applies to the frequency metric C5; Stayton^44^, p. 2146). Stayton’s metrics and their significance are reported in Table 1. We further tested for convergence using the method of Castiglione et al.^47^ implemented in the RRphylo package v.2.4.0^48^. In Brownian-like evolution, the phenotypic distance between two taxa is expected to increase (on average) with the time spent since their cladogenetic divergence. If, instead, it has tended to decrease, this can be taken as evidence that two taxa have converged. This forms the basis of the method developed by Castiglione et al.^47^, where temporal data is thus important. We also applied this method using the first two, the first five, and all axes of the PCoA; the results of these tests and their significance are reported in Table 2.

**Table 1:** Stayton’s convergence metrics for longirostrines (*Marmornectes candrewi*, *Luskhan itilensis*, *Trinacromerum bentonium*) and latirostrines (*Acostasaurus pavachoquensis*, *Plesiopleurodon wellesi*), using the first two, the first five, and all the axes (28) of the PCoA, for each a posterior timescaling method (‘equal’ and ‘minimum branch length’).

	C1	p-value	C2	p-value	C3	p-value	C4	p-value
Longi PCo1-2 equal	0.9110	0.0000	9.4937	0.0000	0.4287	0.0000	0.0751	0.0000
Longi PCo1-5 equal	0.6830	0.0000	8.4503	0.0000	0.2860	0.0000	0.0417	0.0010
Longi all equal	0.2472	0.0010	4.2270	0.0040	0.0849	0.0100	0.0121	0.0390
Lati PCo1-2 equal	0.6937	0.0450	7.8125	0.0519	0.3607	0.0090	0.0729	0.0370
Lati PCo1-5 equal	0.6877	0.0010	9.0724	0.0150	0.2984	0.0010	0.0521	0.0220
Lati all equal	0.1852	0.0559	2.9516	0.1159	0.0590	0.1179	0.0098	0.1658
Longi PCo1-2 mbl	0.9069	0.0010	8.8482	0.0000	0.4569	0.0000	0.0692	0.0020
Longi PCo1-5 mbl	0.6629	0.0000	7.6295	0.0000	0.3016	0.0000	0.0380	0.0010
Longi all mbl	0.2279	0.0000	3.9116	0.0020	0.0928	0.0010	0.0115	0.0090
Lati PCo1-2 mbl	0.6447	0.0500	6.2606	0.0370	0.3018	0.0440	0.0601	0.0649
Lati PCo1-5 mbl	0.6679	0.0020	8.2848	0.0020	0.2926	0.0000	0.0486	0.0140
Lati all mbl	0.1852	0.0160	2.9516	0.0340	0.0673	0.0370	0.0101	0.0989

*Lati* latirostrines, *mbl* minimum branch length, *longi* longirostrines.

**Table 2:** Results of the Castiglione et al. method to assess convergence for longirostrines (*Marmornectes candrewi*, *Luskhan itilensis*, *Trinacromerum bentonium*) and latirostrines (*Acostasaurus pavachoquensis*, *Plesiopleurodon wellesi*), using the first two, the first five, and all the axes (28) of the PCoA, for each a posterior timescaling method (‘equal’ and ‘minimum branch length’).

	*ang.state*	*ang.state.time*	*p.ang.state*	*p.ang.state.time*
Longi PCo1-2 eq	12.67	0.15	0.02	0.01
Longi PCo1-5 eq	47.52	0.56	0.02	0.02
Longi All eq	80.26	0.88	0.02	0.21
Lati PCo1-2 eq	73.65	0.52	0.40	0.22
Lati PCo1-5 eq	50.44	0.36	0.08	0.03
Lati All eq	83.34	0.59	0.17	0.08
Longi PCo1-2 mbl	12.67	0.13	0.01	0.01
Longi PCo1-5 mbl	47.52	0.47	0.01	0.01
Longi All mbl	80.26	0.74	0.02	0.06
Lati PCo1-2 mbl	73.65	0.47	0.40	0.21
Lati PCo1-5 mbl	50.44	0.32	0.09	0.02
Lati All mbl	83.34	0.53	0.18	0.08

*Lati* latirostrines, *mbl* minimum branch length, *longi* longirostrines.

### Disparity through time

We used the dispRity v.1.2.3 package^49^ to compute the differences of total disparity (as a sum of variances, using a bootstrapping procedure, 1000 iterations), per taxonomy (comparing thalassophonean pliosaurids with polycotylids) and per major body plan cluster (latirostrines vs longirostrines); the difference in disparity among each of these pairs was tested using the non-parametric Wilcoxon test (Figs. 2, S2). We computed the disparity through time as a median sum of variance with 50% and 95% confidence intervals via a bootstrapping procedure (1000 bootstraps) for each time bin (Early Jurassic, Mid Jurassic, Late Jurassic, Early Cretaceous and Late Cretaceous) (Fig. 3H). We also compared sums of variance and sums of range, for each time bin to those obtained from 1000 random samples without replacement of the entire dataset (keeping the same number of taxa as the time bin) to evaluate the occurrence of morphological over- or under-dispersion through over time (Table S5).

## Results

### Two recurring morphologies

Our cluster dendrogram recovers a clear divide between two equally diverse morphological groupings (Fig. 2A; PERMANOVA p-value < 0.001). One of the main groups we recover here—which we term ‘longirostrines’—contains early pliosaurids, most polycotylids, and particularly slender-snouted thalassophoneans (*Peloneustes philarchus*, *Luskhan itilensis*, and, to a lesser extent, *Stenorhynchosaurus munozi*). The other main group contains most thalassophonean pliosaurids together with the occultonectian polycotylid *Plesiopleurodon wellesi* (Fig. 2A). Traditionally, the term brevirostrine is used to describe a snout shape opposite to longirostrine; these snouts tend to be short and broad e.g.^50^. Due to the strong constraints imposed by the aquatic medium on fast-swimming predators, their skull shapes retain an elongate rostrum. However, there is a clear separation between elongate crania with slender pre-orbital snouts (“longirostrines”) and elongate crania with robust pre-orbital snouts. For this latter group, we use the term “latirostrine”, as often used for *Crocodylus* and *Alligator* e.g.^51^.

All main subgroups within the longirostrine cluster contain a mixture of polycotylids and pliosaurids. Within the latirostrine cluster, *Plesiopleurodon wellesi* groups with the Cretaceous brachauchenine thalassophonean *Acostasaurus pavachoquensis*. These significantly distinct subgroups mixing early pliosaurids, polycotylids, and thalassophoneans (Table S3) indicate that longirostrines and latirostrines were themselves disparate. Interestingly, the speciose genus *Pliosaurus* occupies several subgroups within the latirostrine cluster, suggesting that this genus may have evolved a disparate array of cranial architectures; both Foffa et al.^13^ and Zverkov et al.^15^ reported a similar degree of disparity in *Pliosaurus*, using dental characters. Nevertheless, our Mantel test finds a significant correlation between the phylogenetic and phenotypic distances (p < 0.001). Indeed, most thalassophoneans occupy the latirostrine cluster while most polycotylids reside in the longirostrine cluster. Yet, pliosaurids and polycotylids each evolved an approximately equal total disparity of craniodental morphologies throughout their entire evolutionary histories (Fig. 2C,D). However, Thalassophonea evolved a significantly greater craniodental disparity; the size of this effect is small, and might be expected given the large difference in lineage longevity (84 Ma for thalassophoneans vs 47 Ma for polycotylids). When postcranial data are added, polycotylids are found to be more disparate than thalassophoneans (Fig. S2). Our Mantel test of the correlation between the phenotypic distance and the time interval separating each taxon indicates that the influence of time is less marked (p = 0.17), as taxa from widely distinct time intervals cluster together, often mixing Jurassic and Cretaceous taxa.

The main features separating the two clusters are the relative width of the snout (wider snouts in latirostrines), the symphysis (shorter symphyses with much lower tooth density in latirostrines), and the tooth crown size (larger in latirostrines) (Fig. S1). Other features, such as the relative length of retroarticular processes, tooth crown shape and postcranial features appear more evenly distributed. Indeed, the addition of postcranial anatomy to the analyses essentially blurs the craniodental signal (compare Figs. 2, 3, S2, S4); the resulting patterns of morphospace occupation appear more influenced by phylogenetic signal and yet form a continuum along the main axes (Figs. S2–S4). This suggest that postcranial data yields a different signal than the craniodental region, but the relatively small amount of postcranial data in our dataset (3 characters out of 11) is not enough to fully test this.

The divide between longirostrine and latirostrine forms is also clear in two dimensional morphospaces using craniodental data (be it PCoA or NMDS; Figs. 3A,C–G, S2–S4), even though the relative eigenvalues of the first two axes of the PCoA are low (14.8% and 9.2% respectively; see Table S3 for a complete list). The non-linear fit of the two-dimensional NMDS suggests that this method better represents the intertaxon distances, with R^2^ = 0.958 (linear fit R^2^ = 0.766). Reassuringly, the patterns of morphospace occupation recovered in two dimensional morphospaces correspond well to the results highlighted above for analysis of data at higher dimensions. *Marmornectes candrewi*, *Peloneustes philarchus*, *Luskhan itilensis*, the Richmond ‘pliosaur’ (an occultonectian polycotylid^11^) and *Trinacromerum bentonianum* are closeby in the morphospace, as do large Late Jurassic-Early Cretaceous thalassophoneans (e.g. *Liopleurodon ferox, Pliosaurus brachydeirus*, *Kronosaurus queenslandicus*). The occultonectian polycotylid *Plesiopleurodon wellesi* positions in between the early thalassophonean *Peloneustes philarchus* and the derived thalassophonean *Acostasaurus pavachoquensis*. With the notable exception of *Luskhan itilensis* and, to a lesser extent, *Stenorhynchosaurus munozi,* Early and middle Cretaceous pliosaurids (*Acostasaurus pavachoquensis*, *Kronosaurus queenslandicus*, *Megacephalosaurus eulerti*, *Sachicasaurus vitae*) fulfilled the latirostrine region by occupying (and slightly expanding in the case of *Sachisasaurus vitae*) the convex hull defined by Middle and Late Jurassic pliosaurids. The situation is different for the derived, Late Cretaceous polycotylids (*Pahasapasaurus haasi*, *Dolichorhynchops osborni*, and *Polycotylus latipinnis*), which markedly expanded the longirostrine morphospace by occupying new zones characterized by even more slender mandibles and longer snouts, in contrast to *Plesiopleurodon wellesi* and Middle Jurassic thalassophonean pliosaurids.

### A bimodal macroevolutionary landscape

Patterns of morphospace occupation, cluster dendrograms, PERMANOVA, and convergence statistics (see Tables 1, 2 and below) depict a congruent picture of a pervasive separation of two main groups that is somewhat independent of phylogeny due to convergent evolution of dietary and functionally important features. This is notably indicated by the transitions between morphotypes involved in (1) the evolutionary origin of Thalassophonea (longirostrine → latirostrine); (2) the longirostrine thalassophonean *Luskhan itilensis* (latirostrine → longirostrine); and (3) the occultonectian polycotylid *Plesiopleurodon wellesi* (longirostrine → latirostrine).

According to our protocol (see “Methods” section; Fig. 1), this concordance makes it appropriate to approximate the macroevolutionary landscape using the density of phenotypes. The kernel density estimator creates a new dimension that translates the intensity of occupation of each region of morphospace. If plotted as a third dimension on the two-dimensional morphospaces, this density value approximates a macroevolutionary landscape by hypothesising that frequently recorded phenotypes (peaks) represent optimal morphologies, whereas the valleys represent morphologies rarely or never occupied in the sample. This method recovers two main peaks: the tallest and narrowest representing the longirostrine peak, whereas the latirostrine taxa form a slightly broader and lower peak (Fig. 3B). Moreover, no species yet discovered records an unambiguously intermediate morphology, resulting in a trough between the main peaks of the craniodental macroevolutionary landscape (Fig. 3B). Although such intermediates might have existed during evolutionary transitions, they were sufficiently rare to have thus far escaped detection in the fossil record, suggesting that transitions occurred rapidly under selection or that the transitions were not gradual because of co-evolution of character complexes. As was the case for the cluster dendrogram analysis, adding postcranial data reduced interclade convergence and resulted in macroevolutionary landscapes where individualised peaks were difficult to discern (Figs. S2–S4).

Convergence statistics unanimously identify key longirostrine taxa as examples of convergent evolution: the interclade group *Marmornectes candrewi* + *Luskhan itilensis* + *Trinacromerum bentonianum* is regarded as significantly convergent for every Stayton^44^ metric, no matter the number of PCoA axes used or the method used to timescale the phylogenetic tree (Table 1). These taxa are also identified as unambiguously convergent by the “search.conv” method of Castiglione et al.^47^ when using the first two and first five PCoA axes, but not when all of them are used (Table 2). Stayton’s metrics also recover the *Plesiopleurodon wellesi* + *Acostasaurus pavachoquensis* latirostrine pair as significantly convergent, but only when the first two or five PCoA axes are used; these taxa are not significantly convergent (at alpha = 0.05) when all PCoA axes are used (Table 1) using the ‘equal’ timescaled tree. However, all Stayton’s metrics identify the same pair as significantly convergent, for any number of PCoA axes, based on the tree timescaled using minimum branch lengths (Table 1). The search.conv method^47^ recovers the *Plesiopleurodon wellesi* + *Acostasaurus pavachoquensis* pair as significantly convergent when applied to the first five PCoA axes (Table 2).

### Patterns of disparity through time

The total disparity evolved by the member of each craniodental ecomorphological cluster (latirostrines|longirostrines) is quite similar (Fig. 2C,D); longirostrines are significantly more disparate, incorporating several members of each clade, but the relative importance of this difference is small. The disparity through time (Fig. 3H) appears fairly stable, with lower values during the Late Jurassic when no longirostrine plesiosaurians are recorded, and gradually increasing across the Jurassic-Cretaceous transition and then during the Cretaceous (Fig. 3H). The disparity increase we document across the Jurassic-Cretaceous transition is consistent with the patterns recovered on pliosaurid teeth^15^. Our results indicate that short-necked plesiosaurians were never significantly over- or underdispersed morphologically throughout their evolutionary history (Table S5). Nevertheless, the morphological disparity during the Late Jurassic is very close to underdispersion, resulting from the *Pliosaurus*-dominated assemblage lacking longirostrine taxa, as already noted by Foffa et al. on teeth^13^. On the contrary, the Late Cretaceous assemblages are close to over-dispersion, combining the last thalassophonean latirostrines (*Brachauchenius lucasi*, *Megacephalosaurus eulerti*), a polycotylid latirostrine (*Plesiopleurodon wellesi*), as well as a series of derived polycotylids (notably *Pahasapasaurus haasi* and *Dolichorhynchops* spp.) that have clearly expanded the longirostrine body plan towards novel morphologies (Fig. 3).

## Discussion

The pattern of craniodental morphospace occupation of short-necked plesiosaurians is bimodal, being composed of two principal and recurring morphotypes (longirostrines and latirostrines), which transcend phylogeny. Compared to latirostrine forms, longirostrine plesiosaurians have notably slender snouts with longer symphyses, smaller teeth, and smaller skulls (Figs. 2, S1). These taxa also usually lack the strong apical wear and spalling seen in latirostrine taxa^52^ such as in *Liopleurodon*, *Pliosaurus*, ‘*Polyptychodon’*, and *Brachauchenius*^15, 53–55^. On one hand, longirostrine taxa thus likely have a reduced range of prey types they can effectively process compared to latirostrines, both in terms of size and hardness (internal or external), having a generally weaker bite force and less resistance to twisting^14, 56^ (but see and Da Silva et al.^57^ and McCurry et al.^50^ for discussions on how the long-snouted river dolphin *Inia* is able to crush small turtles using its most distal teeth). On the other hand, a more slender snout means that a smaller volume of water needs to be displaced during jaw closure, facilitating the capture of small and fast prey that might otherwise be expelled by the water flow^58^. These features, along with tooth size and shape, have evident functional implications and form a guild-defining character complex (Fig. S1) that possibly explains the absence of true morphological intermediates in our dataset. This, in turn, likely played a role in reducing the number of transitions (hence resulting in a strong phylogenetic signal) via niche conservatism^59^. Transitions were moderately rare, but involved a significant change of morphology through convergent evolution (Figs. 2, 3, Tables 1, 2), suggesting the existence of a pervasive macroevolutionary landscape channelling the craniodental evolution of short-necked plesiosaurians.

Our results cast doubt on the existence of a single “pliosauromorph” optimal morphology. We recover the polycotylid *Plesiopleurodon wellesi* as unambiguously convergent with large, latirostrine, and coeval^60^ pliosaurids; all other polycotylids as well the pliosaurids *Hauffiosaurus zanoni*, *Peloneustes philarchus*, *Marmornectes candrewi*, *Luskhan itilensis*, and *Stenorhynchosaurus munozi* clearly possess a distinct common morphology. The resemblance of taxa grouped as “pliosauromorphs” is only superficial, resulting from iterative ecological convergence of weakly related lineages rather than a single optimal craniodental architecture driving the diversification of a monophyletic group. This is also substantiated by the signal yielded by postcranial anatomy, which differs from the craniodental pattern. This suggests that craniodental and postcranial regions of short-necked plesiosaurians were influenced by distinct evolutionary pressures, which again undermines the concept of a singular “pliosauromorph” body architecture optimum. As such, our results support the claims of O’Keefe^10^ that a unique “pliosauromorph” morphology does not exist; this term should be restricted to gross body proportions in a broad-brush view of the macroevolution of marine tetrapods.

Our method also allows detection of perturbations of the landscape over time. The two main morphotypes were already present by the Middle Jurassic (Fig. 3), thanks to the co-occurrence of primitive, longirostrine pliosaurids and the earliest latirostrine thalassophoneans^41^. Subsequent evolution repeatedly explored these two regions of the morphospace, suggesting the existence of strong and durable constraints. While the Late Jurassic radiation of the cosmopolitan genus *Pliosaurus*^61–64^ expanded the latirostrine space; this period also marks a peculiar chapter in plesiosaurian history with no representatives in the longirostrine group (and a lower total disparity; Fig. 3, Table S3). This absence took place at a time when long-snouted ichthyosaurs and thalattosuchians (marine crocodylomorphs) were particularly diverse^13, 16, 65, 66^. The Late Cretaceous marks a restructuring of the evolutionary patterns that influenced the earlier evolution of short-necked plesiosaurians. The slender-snouted and supposedly fast-swimming^67^ polycotylids of the Late Cretaceous colonised entirely new regions in the morphospace, and the extinction of both pliosaurids and early polycotylids marks the definite end of latirostrine plesiosaurians after the Turonian. As a result, the macroevolutionary landscape of short-necked plesiosaurians collapsed to a unimodal distribution. This profound alteration of evolutionary dynamics probably resulted from the equally-profound changes in the structure of marine ecosystems that took place during the earliest Late Cretaceous. Indeed, several groups of cephalopods declined and ichthyosaurs went extinct, possibly as a consequence of environmental volatility^16, 68–70^, while mosasauroids, elasmosaurids, teleosts, and selachians abruptly radiated^71–77^, among other biotic and climatic events e.g.^78^. Pliosaurids became extinct at or close to the Turonian-Coniacian boundary^79, 80^, concomitant with the radiation of large-bodied mosasauroids^72, 79^. Evolving in ichthyosaur-free but shark- and mosasauroid-packed oceans, Late Cretaceous plesiosaurians colonised new regions of the morphospace, possibly filling the gap(s) left by the demise of ichthyosaurs and diversifying the long-necked body plan^77, 81, 82^. This suggests that restructuring of the upper levels of oceanic ecosystems by the beginning of the Late Cretaceous fundamentally altered the macroevolutionary landscape of plesiosaurians.

Macroevolutionary landscapes are rarely investigated in a multivariate framework as phylogenetic relatedness^44, 83^ and extinction effects^84, 85^ might drive morphospace occupation concomitantly to convergent evolutionary processes. When coupled with thorough tests for convergence, ecomorphological traits can be used to reveal the overarching possibilities and constraints of evolution^46, 86, 87^ (Fig. 1). This study is the first to use a rigorous quantitative and phylogenetically-explicit framework to assess ecomorphological diversity and patterns of niche occupation through time in extinct marine reptiles, building upon previous attempts at quantification^10, 13–17^. Our protocol is generalizable to any set of taxa for which independent ecomorphological and cladistic data can be gathered.

## Conclusions

We develop a protocol that approximates macroevolutionary landscapes based on phenotypic data to explore patterns of convergence and constraint within clades for which independent ecomorphological and cladistic data can be gathered. We apply this protocol to short-necked plesiosaurians, providing the first phylogeny-explicit quantitative assessment of trophic diversity in extinct marine reptiles. We reveal a pervasive bimodal landscape and that was established early in the history of Plesiosauria; one peak represents latirostrine taxa with robust skulls while the other represents longirostrine forms, supported by guild-defining character complexes. Although phyletic transitions from one peak to another are rare, the lineages doing so were found to be statistically convergent, suggesting the existence of a strong evolutionary pressure channelling the craniodental evolution of short-necked plesiosaurians. Only a profound reorganization of marine ecosystems during the early Late Cretaceous markedly altered this long-lasting pattern, collapsing the macroevolutionary landscape to unusual longirostrine forms.

## Supplementary information

Supplementary Information.

## Data Availability

All the 3D models we used will be provided in open access as .ply files on Morphosource (P1018; http://www.morphosource.org/Detail/ProjectDetail/Show/project_id/1018) upon acceptance. All the datasets and scripts we use are provided as Supplementary Files.
