# Supplementary material for: The macroevolutionary landscape of short-necked plesiosaurians
Source: Sci Rep. 2020 Oct 2;10:16434. doi: 10.1038/s41598-020-73413-5 (PMC7532190; doi:10.1038/s41598-020-73413-5)
Supplement: Supplementary file 1 — Supplementary Information. [file 41598_2020_73413_MOESM1_ESM.pdf]

THE MACROEVOLUTIONARY LANDSCAPE OF SHORT-NECKED PLESIOSAURIANS

SUPPLEMENTARY INFORMATION

Valentin Fischer[1], Jamie A. MacLaren[1], Laura C. Soul[2], Rebecca F. Bennion[1,3], Patrick S. Druckenmiller[4] & Roger B.J. Benson[5]

[1] Evolution & Diversity Dynamics Lab, Université de Liège, Liège, Belgium.
[2] Department of Paleobiology, Smithsonian Institution, P.O. Box 37012, Washington, DC 20013-7012, Unites State of America.
[3] OD Earth and History of Life, Institut royal des Sciences Naturelles de Belgique, Rue Vautier, 29, 1000 Brussels, Belgium.
[4] University of Alaska Museum and Department of Geosciences, University of Alaska Fairbanks, 1962 Yukon Drive, Fairbanks, AK 99775, Unites State of America.
[5] Department of Earth Sciences, University of Oxford, Oxford OX1 3AN, United Kingdom.

Corresponding author:
Valentin Fischer
B18, 14 Allée du 6 Août, 4000 Liège, Belgium.
Email address: v.fischer@uliege.be

STRUCTURE

SUPPLEMENTARY PHYLOGENETIC INFORMATION 2

SUPPLEMENTARY TABLES 3

SUPPLEMENTARY FIGURES........ 9

REFERENCES........ 15

SUPPLEMENTARY PHYLOGENETIC INFORMATION

We modified the matrix of Fischer et al. [1] by adding six species: *Rhaeticosaurus mertensi* [2], *Acostasaurus pavachoquensis* [3], *Pliosaurus patagonicus* [4], *Pliosaurus almanzaensis* [5], *Sachicasaurus vitae* [6], and *Kronosaurus boyacensis* [7]. We also modified the scores of *Stenorhynchosaurus munozi* according to [8], except for the following four characters, for which we retained the scores of [1,9]:

- #49. The parietal vault is poorly preserved and the character scores appears as ambiguous from the data provided in [8]. We scored this character as "?".
- #56. We think that the bone interpreted as a jugal in [8] contains parts of the squamosal, which is especially evident in Figure 3C of [8], where the bone interpreted as a jugal forms most of squamosal arch. If true, this would mark a clear departure from the morphology observed other derived pliosaurids [9–11]. Moreover, the jugal-squamosal connection appears to be broken off. For these reasons, we conservatively scored this character as "?".
- #127. We kept the score "1", because the dorsal portion of the surangular still appears blade-like (Figure 2A in [8]).
- #130 We kept the score "0", because the dorsal surface of the articular does not seemingly form a concave surface (Figures 2 and 3 in [8]).

SUPPLEMENTARY TABLES

Table S1. Taxonomic sampling and completeness ratio. *Manemergus anguirostris* was removed prior to the analyses because of the (very) young ontogenetic stage of the type and only specimen [1,12]; we decided to keep the data here for further use. Taxa in grey did not pass the 50% completeness threshold and were also removed prior to the analyses. Surface scanned taxa are marked with an *; see supplementary information to download the 3D models.

Taxon	Clade	Completeness	Source of data
Hauffiosaurus longirostris	Pliosauridae	36.4%	[13,14]; measurements from photographs of the holotype
Hauffiosaurus tomistomimus	Pliosauridae	18.2%	[14] and measurements on MMUM LL8004
Hauffiosaurus zanoni	Pliosauridae	72.7%	[15]; measurements on photos of HAUF 7 (uncatalogued at the time)
Marmornectes candrewi	Pliosauridae	72.7%	[16]; measurements on photos of the holotype
Peloneustes philarchus	Thalassophonea	90.9%	Measurements on GPIT 03182
Simolestes vorax	Thalassophonea	100%	[17]; measurements on photos from Andrews [18] and Noé [19] (NHMUK R3319)
Pliosaurus westburyensis	Thalassophonea	54.5%	[20,21] and measurements on photos BRSMG CC332
Pliosaurus carpenteri	Thalassophonea	54.5%	[21,22] and measurements on photos of BRSMG Cd6172
Pliosaurus brachydeirus	Thalassophonea	45.4%	Measurements on OUMNH J.9245, and estimations from Knutsen [22]
Pliosaurus kevani	Thalassophonea	72.7%	[11] and measurements on photos of DORCM G.13,675
Pliosaurus andrewsi	Thalassophonea	54.5%	[19] and measurements from photos NHMUK R3891
Pliosaurus brachyspondylus	Thalassophonea	72.7%	[22–24] and measurements on CAMSM J 35991
Liopleurodon ferox	Thalassophonea	81.8%	Measurements on GPIT 03184 (previously 1754/2)
*Kronosaurus queenslandicus**	Thalassophonea	72.7%	Data from McHenry [25] on QM F10113 and QM

			F18827 (only for teeth) and Holland [26] (KK F0630, for mandible proportions). Measurements from photos and laser scans of QM F10113 (0.8mm resolution) and a cast of MCZ 1285 held at QM (0.2mm resolution)
*Megacephalosaurus eulerti**	Thalassophonea	63.6%	Measurements a white light scan of FHSM VP-321 (0.5 to 1mm resolution; provided by Chase Shelburne) and [27] for additional tooth crown data
*Brachauchenius lucasi**	Thalassophonea	63.6%	Measurements from a laser scan of the holotype (USNM 4989) (0.5mm resolution)
Stenorhynchosaurus munozi	Thalassophonea	63.6%	[8,28] and measurements from photos
Anguanax zignoi	Thalassophonea	27.3%	[29,30] and measurements from photos
Makhaira rossica	Thalassophonea	18.2%	[31] and measurements on YKM 68249/1-10
Luskhan itilensis	Thalassophonea	72.7%	[9] and measurements on YKM 68344/1_262
Acostasaurus pavachoquensis	Thalassophonea	72.7%	[3] and measurements from photos of UNDG R-1000
Pliosaurus patagonicus	Thalassophonea	0.00%	[4] and measurements from photos of MLP 80-V-29-1
Pliosaurus almanzaensis	Thalassophonea	36.4%	[5] and measurements from photos of MOZ 3728P
Sachicasaurus vitae	Thalassophonea	72.7%	[6] and measurements from photos of MP111209-1
Kronosaurus boyacensis	Thalassophonea	27.3%	Data from "El fosil" Hampe 1992; measurements from photos
Edgarosaurus muddi	Polycotylidae	81.8%	[32] and measurements from photos of MOR 751
*Plesiopleurodon wellesi**	Polycotylidae	72.7%	Measurements on a laser scan of CM 2815 (0.3mm resolution)
Richmond 'pliosaur'*	Polycotylidae	81.8%	Measurements on a laser scan of QM F1609 (0.3mm resolution)
Palmulasaurus quadratus	Polycotylidae	9.1%	[33] and humerus/femur ratio from [34] and

			measurements from photos of MNA V9442
Pahasapasaurus haasi	Polycotylidae	54.5%	[35] and measurements from photos of AMM 98.1.1
Polycotylus latipinnis	Polycotylidae	81.8%	[36] and measurements from photos SDSM 23020) for cranium; [17] for neck/skull ratio (using specimen YPM 1125)
Thililua longicollis	Polycotylidae	63.6%	[1,37] and measurements on MHNGr.PA. 11710
*Trinacromerum bentonianum**	Polycotylidae	72.7%	Measurements from a laser scan (resolution 0.3 mm) of KUVP 5070; [17] for neck/skull ratio; [38] for symphysial teeth
*Dolichorhynchops osborni**	Polycotylidae	90.9%	Measurements from a laser scan (resolution 0.3 mm) of KUVP 1300; [38] for symphysial teeth
*Dolichorhynchops bonneri**	Polycotylidae	81.8%	Measurements from a laser scan (resolution 0.3 mm) of KUVP 40001. Symphysial teeth data from [38] ; limb data from [39]
Eopolycotylus rankini	Polycotylidae	18.2%	[33] and measurements from photos of MNA V9445
Dolichorhynchops tropicensis	Polycotylidae	81.8%	[34] and measurements from photos of MNA V10046; McKean pers. comm. Dec. 2018 for coronoid; Gillette pers. comm. Dec. 2018 for retroarticular
Dolichorhynchops herschelensis	Polycotylidae	54.5%	[40] and measurements from photos of RSM P2310.1
Sulcusuchus erraini	Polycotylidae	0.00%	[41] and measurements from photos of MPEF 650
Mauriciosaurus fernandezi	Polycotylidae	54.5%	[42] and measurements from photos of CPC RFG 2544 P.F.1)
Manemergus anguirostris	Polycotylidae	90.9%	[12] and measurements on SMNK 386

Table S2. Morphometric ratios and measurements used to quantify Baupläne, with description of function or characterisation justification.

Name	Explanation	Completeness
**HL_mandible**	Height of the mandible (at the level of the coronoid process) ÷ length of mandible. Characterises maximum aspect ratio for jaw; proxy for the maximum dorsoventral flexural stiffness of the jaw[43–45].	57.5%
**Retro_coro**	Length of retroarticular process ÷ height of the mandible at the level of the coronoid process. Characterises the leverage to open the jaw against the drag of the water [46–48].	50%
**Snout_width**	Skull width just anterior to the orbit ÷ mandible length. Characterises skull shape and is related to (i) resistance to lateral shaking[49], and (ii) to the volume of water needed to be expelled to close the jaws [50].	45%
**Rel_snout**	Snout length ÷ mandible length. Characterises snout shape and is related to (i) resistance to lateral shaking[49], (ii) to the volume of water needed to be expelled to close the jaws [50], and (iii) to the amount of drag during swimming [51].	57.5%
**Rel_Symphysis**	Symphyseal length ÷ mandible length. Characterises the shape of the snout and the mechanical response of the anterior jaw to dorsoventral, mediolateral and rotational loads during biting [44,45,52].	77.5%
**Symph_teeth_density**	Number of symphyseal teeth ÷ symphysis length. Proxy for the preferential use of the anterior jaw for prey capture [45].	60%
**Crown_height**	Maximum absolute crown height. Absolute crown size has been shown to be a major determinant of diet in modern cetaceans [53].	67. 5%
**Crown_shape**	Crown height ÷ crown basal diameter. Characterises tooth shape (slender vs. broad); proxy for bending resistance of the tooth [45,54–56].	70%
**Hum/Fem**	Humerus proximodistal length ÷ femur proximodistal length. Characterises the appendicular body plan. Humeri and femora are known to reduce in length with increased employment for swimming [57,58]; the length of the humerus and femur relative to one another may therefore be considered as a proxy for fore-limb driven, hind-limb driven or equally driven underwater locomotion.	57.5%
**Mand/fem**	Mandible length ÷ femur proximodistal length. Animals with relatively long limbs and small heads will swim, accelerate, and catch food in distinct manner to animals with relatively large heads and small limbs [59,60]. This character is therefore a proxy for elongation of limbs relative to skull size.	50%
**Neck/skull**	Neck length ÷ mandible length. Characterises the general body plan; proxy for potential feeding arc and reach of cranium away from the trunk [61].	45%

Table S3. PERMANOVA p-values, testing the strength of the main clusters (cut =2) and increasingly smaller subclusters (cut = 3:10).

Cut value	PERMANOVA p-value
**2**	<0.001 (***)
**3**	<0.001 (***)
**4**	<0.001 (***)
**5**	<0.001 (***)
**6**	<0.001 (***)
**7**	<0.01 (**)
**8**	<0.05 (*)
**9**	0.1359
**10**	0.1688

Table S4. Results of the principal coordinates analysis (PCoA) on craniodental data.

	Eigenvalues	Corrected eigenvalues	Relative corrected eigenvalues	Cumulative relative eigenvalues
**1**	102.193121771145	463.869462205815	0.148492965411724	0.148492965411724
**2**	61.1457772100602	286.698653395902	0.0917774000898045	0.240270365501528
**3**	37.412108106037	230.906291332669	0.0739172606214767	0.314187626123005
**4**	37.1438087569245	218.014953030361	0.0697905111615476	0.383978137284553
**5**	24.1970034832429	184.980045187141	0.0592154424678333	0.443193579752386
**6**	17.4956260661365	154.380339782146	0.0494199259130125	0.492613505665399
**7**	14.6075672498017	139.40377519477	0.0446256579810724	0.537239163646471
**8**	10.3880323787846	123.366667471945	0.0394918910996198	0.576731054746091
**9**	4.87235481758962	105.516997690975	0.0337778904736844	0.610508945219775
**10**	3.44554160087047	98.5462618389466	0.0315464324405401	0.642055377660315
**11**	2.47363171827753	95.9988649635975	0.0307309648426034	0.672786342502919
**12**	1.33856470420492	90.1189258073412	0.0288486904682545	0.701635032971173
**13**	1.11702012312488	85.0453908259296	0.0272245605871294	0.728859593558302
**14**	0.0565502248815406	82.3023067639064	0.0263464499979853	0.755206043556288
**15**	0	78.2362022814413	0.0250448167552968	0.780250860311585
**16**	-0.354687044831845	76.2641335132217	0.024413522041486	0.804664382353071
**17**	-0.489879979068597	74.4051616679499	0.0238184316886772	0.828482814041748
**18**	-0.680875139407188	72.9336824358365	0.023347384696967	0.851830198738715
**19**	-0.861558503234132	70.3399447859105	0.0225170825828634	0.874347281321578

**20**	-1.55745510920335	69.5584661827946	0.0222669172138593	0.896614198535437
**21**	-1.83599166025336	66.0102568940532	0.0211310715458104	0.917745270081248
**22**	-2.24507794773183	63.2571664406893	0.0202497577306636	0.937995027811911
**23**	-4.04686739313769	56.3910713738424	0.0180517970965951	0.956046824908506
**24**	-5.14765636630927	53.5068377716017	0.017128501998659	0.973175326907165
**25**	-6.44220248625217	35.8548873857984	0.0114777949104533	0.984653121817619
**26**	-8.79691827973208	30.6130863821715	0.00979980004649877	0.994452921864117
**27**	-13.9451315515094	17.3282292839322	0.00554707813588232	1
**28**	-21.2566818323273	0	0	1
**29**	-22.5385094591177	0	0	1

Table S5. Test of disparity overdispersion over time. The values of the sum of range (SoR) and sum of variance (SoV) for each time bin (Early Jurassic [EJ], Mid Jurassic [MJ], Late Jurassic [LJ], Early Cretaceous [EK], and Late Cretaceous [LK]) always fall within the mean ± 2*standard deviation of our bootstrapping procedure using the entire dataset (1000 random samples without replacement of all taxa, with the same bin size as the time bin to which it is compared). Hence, no time bin shows a notable under- or overdispersion of morphological disparity, even though the Late Jurassic bin is very close to the underdispersion threshold.

	EJ	MJ	LJ	EK	LK
**SoR**	NA	117.43	113.76	131.50	171.49
**Boot_SoR_mean-2sd**	NA	113.25	112.95	124.04	158.24
**Boot_SoR_mean+2sd**	NA	127.68	127.96	138.88	173.56
**SoV**	NA	104.82	94.18	111.52	108.51
**Boot_SoV_mean-2sd**	NA	94.14	94.05	96.09	102.33
**Boot_SoV_mean+2sd**	NA	128.61	128.45	126.83	120.71

SUPPLEMENTARY FIGURES

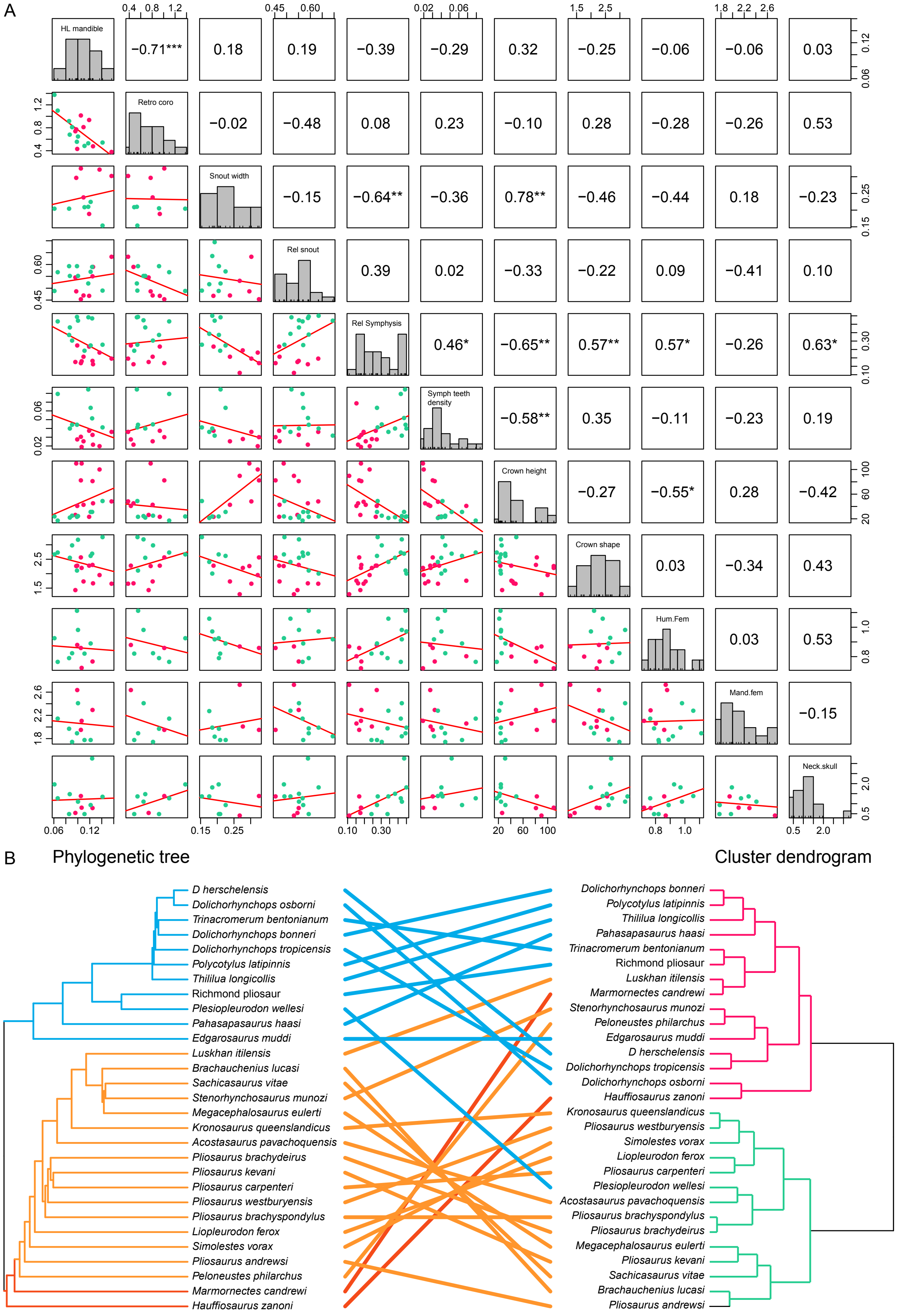

Figure S 1. A, Histograms, pairwise distribution, and correlation of the Bauplan variables used here. The lower panels denote the distribution of the data, and each dot is coloured with respect to the major clusters: longirostrines in aquatic green and latirostrines in dark pink. The upper panels indicate the pairwise correlation (Pearson's correlation coefficient); asterisks indicate significance at alpha=0.05(*) and 0.01(**). B, Tanglegram comparing phylogeny (one randomly sampled most parsimonious tree) with the hierarchy of the cluster dendrogram, craniodental dataset. The packages psych v1.8.12 [62] and dendextend v.1.13.2. [63] in the R v3.6.2 statistical environment [64] (https://www.r-project.org) were used to produce this figure.

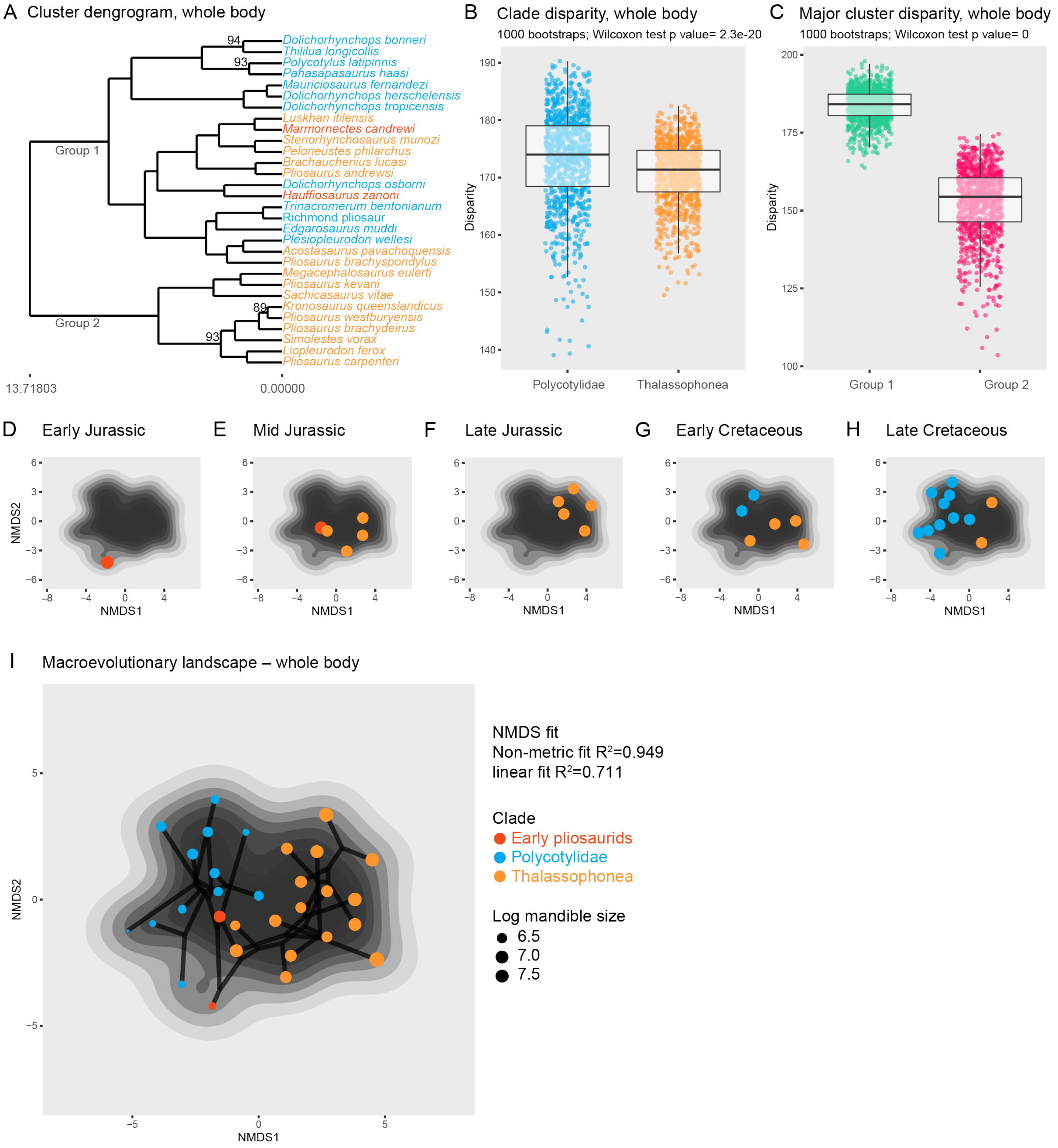

Figure S 2. Results of whole body analyses. A, Cluster dendrogram using the entire dataset. Values of node support (approximate unbiased p-value) are indicated when below 97%. B, comparison of total disparity per taxonomy (Polycotylidae | Thalalassophonea). C, comparison of total disparity per Bauplan cluster (group 1 | group 2). D-I, Morphospace occupation over time and density using NMDS of the whole dataset. D, Early Jurassic occupation superimposed on the final landscape. E, Middle Jurassic occupation superimposed on the final landscape. F, Late Jurassic occupation superimposed on the final landscape. G, Early Cretaceous occupation superimposed on the final landscape. H, Late Cretaceous occupation superimposed on the final landscape. I, all taxa with superimposed phylogeny, using a randomly-sampled most parsimonious tree. The packages ggplot2 v3.3.1 [65], ggdendro v0.1-20

[66] , dendextend v.1.13.2. [63], ggrepel v0.8.1 [67], gridextra v2.3. [68] and plotly v4.9.1 [69] in the R v3.6.2 statistical environment [64] (https://www.r-project.org) were used to produce this figure.

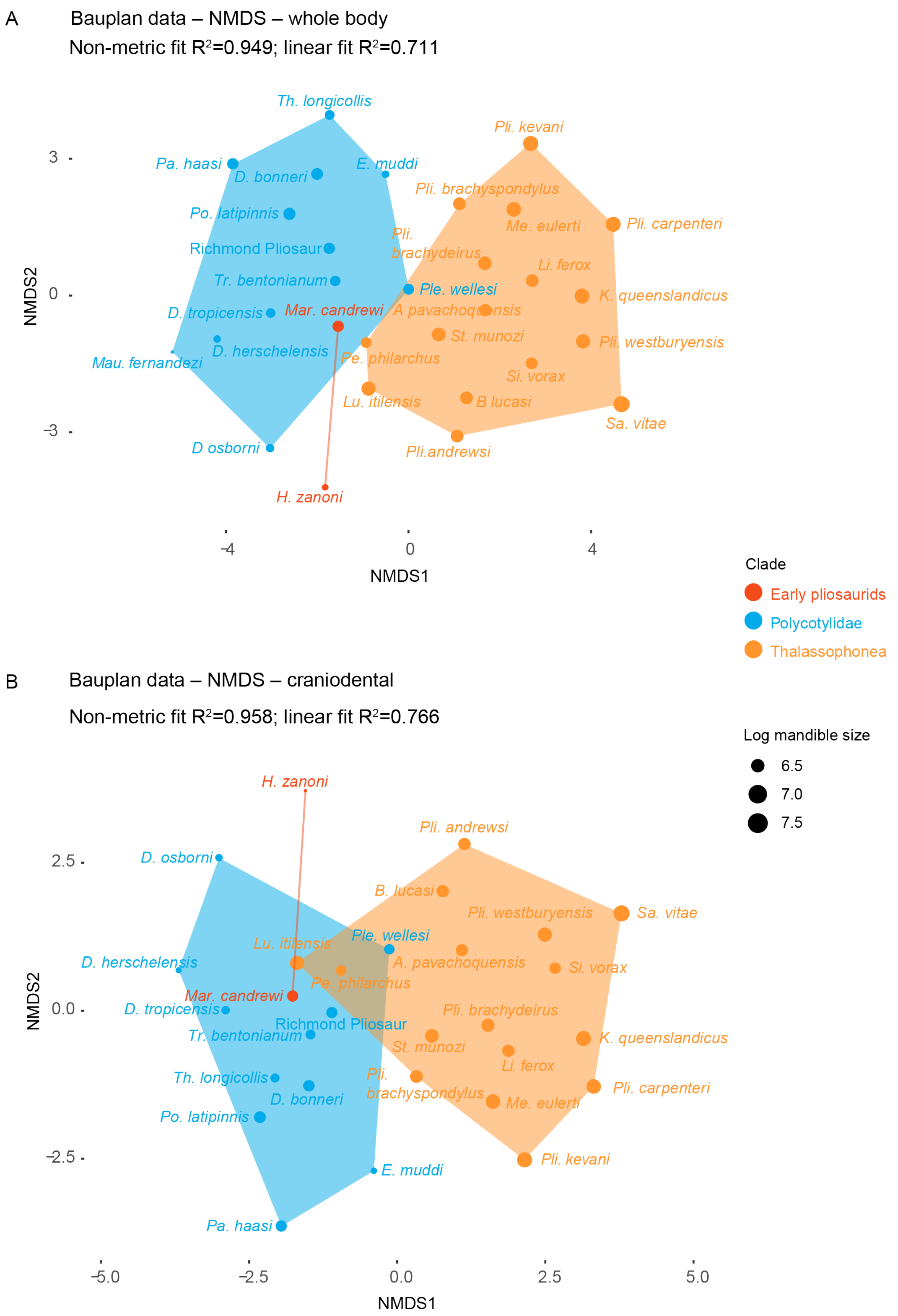

Figure S 3. Morphospaces using NMDS of the whole dataset (A) and the craniodental dataset (B). The size of each data is proportional to the log10 of mandible size. The package ggplot2 v3.3.1 [65] in the R v3.6.2 statistical environment [64] (https://www.r-project.org) was used to produce this figure.

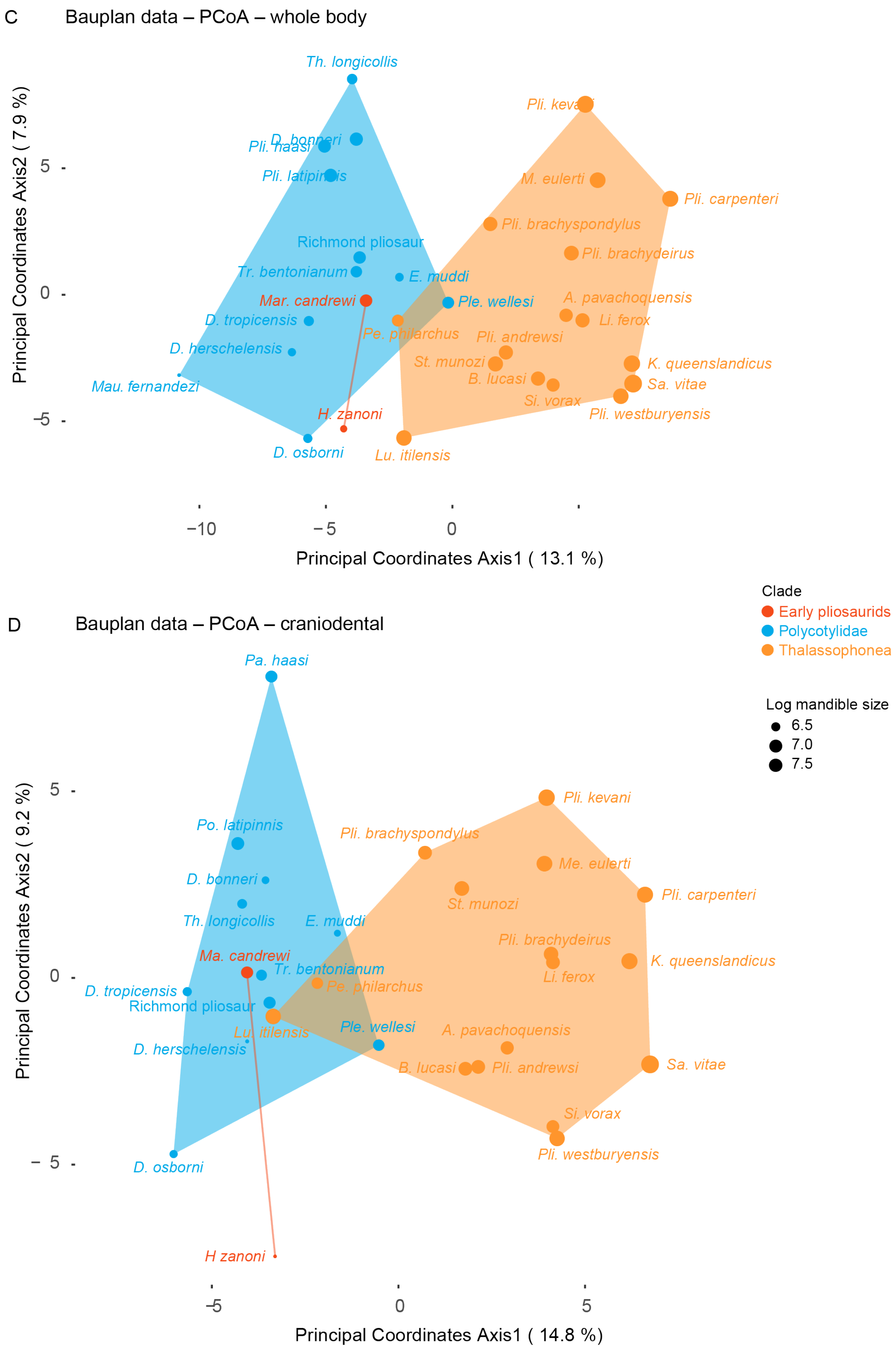

Figure S 4. Morphospaces using the first two axes of the PCoA of the whole dataset (A) and the craniodental dataset (B). The size of each data is proportional to the log10 of mandible size. The package ggplot2 v3.3.1 [65] in the R v3.6.2 statistical environment [64] (https://www.r-project.org) was used to produce this figure.

References

1. Fischer, V., Benson, R. B. J., Druckenmiller, P. S., Ketchum, H. F. & Bardet, N. The evolutionary history of polycotylid plesiosaurians. *R. Soc. Open Sci.* **5**, (2018).
2. Wintrich, T., Hayashi, S., Houssaye, A., Nakajima, Y. & Sander, P. M. A Triassic plesiosaurian skeleton and bone histology inform on evolution of a unique body plan. *Sci. Adv.* **3**, e1701144 (2017).
3. Gómez-Pérez, M. & Noè, L. F. Cranial anatomy of a new pliosaurid *Acostasaurus pavachoquensis* from the Lower Cretaceous of Colombia, South America. *Palaeontogr. Abteilung A* **310**, 5–42 (2017).
4. Gasparini, Z. & O'Gorman, J. P. A New Species of *Pliosaurus* (Sauropterygia, Plesiosauria) from the Upper Jurassic of Northwestern Patagonia, Argentina. *Ameghiniana* **51**, 269–283 (2014).
5. O'Gorman, J. P., Gasparini, Z. & Spalletti, L. A. A new *Pliosaurus* species (Sauropterygia, Plesiosauria) from the Upper Jurassic of Patagonia: new insights on the Tithonian morphological disparity of mandibular symphyseal morphology. *J. Paleontol.* 1–14 (2018) doi:10.1017/jpa.2017.82.
6. Páramo-Fonseca, M. E., Benavides-Cabra, C. D. & Gutiérrez, I. E. A new large pliosaurid from the barremian (Lower cretaceous) of sáchica, boyacá, colombia. *Earth Sci. Res. J.* **22**, 223–238 (2018).
7. Hampe, O. Ein grosswüchsiger pliosauride (Reptilia, Plesiosauria) aus der Unterkreide (oberes Aptium) von Kolumbien. *Cour. Forschungsintitut Senckenb.* **145**, 1–32 (1992).
8. Páramo-Fonseca, M. E., Benavides-Cabra, C. D. & Gutiérrez, I. E. A new specimen of *Stenorhynchosaurus munozi* Pliosauridae), from the Barremian of Colombia : new morphological features and ontogenetic implications. *J. Vertebr. Paleontol.* **39**, 1–16 (2019).
9. Fischer, V. *et al.* Plasticity and convergence in the evolution of short-necked plesiosaurs. *Curr. Biol.* **27**, 1667–1676 (2017).
10. Schumacher, B. A., Carpenter, K. & Everhart, M. J. A new Cretaceous Pliosaurid (Reptilia, Plesiosauria) from the Carlile Shale (middle Turonian) of Russell County, Kansas. *J. Vertebr. Paleontol.* **33**, 613–628 (2013).
11. Benson, R. B. J. *et al.* A giant pliosaurid skull from the Late Jurassic of England. *PLoS One* **8**, e65989 (2013).
12. Buchy, M.-C., Métayer, F. & Frey, E. Osteology of *Manemergus anguirostris* n.gen. et sp., a new plesiosaur (Reptilia, Sauropterygia) from the Upper Cretaceous of Morocco. *Palaeontographica* **272**, 97–120 (2005).
13. White, T. E. Holotype of *Plesiosaurus longirostris* Blake and classification of the plesiosaurs. *J. Paleontol.* **14**, 451–467 (1940).
14. Benson, R. B. J., Ketchum, H. F., Noè, L. F. & Gómez-Pérez, M. New information on *Hauffiosaurus* (Reptilia, Plesiosauria) based on a new species from the Alum Shale member (Lower Toarcian: Lower Jurassic) of Yorkshire, UK. *Palaeontology* **54**, 547–571 (2011).
15. Vincent, P. A re-examination of *Hauffiosaurus zanoni*, a pliosauroid from the Toarcian (Early Jurassic) of Germany. *J. Vertebr. Paleontol.* **31**, 340–351 (2011).
16. Ketchum, H. F. & Benson, R. B. J. A new pliosaurid (Sauropterygia, Plesiosauria) from the Oxford Clay Formation (Middle Jurassic, Callovian) of England: evidence for a gracile, longirostrine grade of Early–Middle Jurassic

pliosaurids. *Spec. Pap. Palaeontol.* **86**, 109–129 (2011).
17. O'Keefe, F. R. The evolution of plesiosaur and pliosaur morphotypes in the Plesiosauria (Reptilia: Sauropterygia). *Palaeobiology* **28**, 101–112 (2002).
18. Andrews, C. W. *A descriptive catalogue of the marine reptiles of the Oxford clay. Based on the Leeds Collection in the British Museum (Natural History), London. Part I.* (Order of the Trustees of the British Museum, 1910).
19. Noe, L. F. A taxonomic and functional study of the Callovian (Middle Jurassic) Pliosauroidea (Reptilia, Sauropterygia). (University of Derby, 2001).
20. Taylor, M. A. & Cruickshank, A. R. I. Cranial Anatomy and Functional Morphology of *Pliosaurus brachyspondylus* (Reptilia: Plesiosauria) from the Upper Jurassic of Westbury, Wiltshire. *Philos. Trans. R. Soc. B Biol. Sci.* **341**, 399–418 (1993).
21. Sassoon, J., Noè, L. F. & Benton, M. J. Cranial anatomy, taxonomic implications and palaeopathology of an Upper Jurassic pliosaur (Reptilia: Sauropterygia) from Westbury, Wiltshire, UK. *Palaeontology* **55**, 743–773 (2012).
22. Knutsen, E. M. A taxonomic revision of the genus *Pliosaurus* (Owen, 1841a) Owen, 1841b. *Nor. J. Geol.* **92**, 259–276 (2012).
23. Tarlo, L. B. *Pliosaurus brachyspondylus* (Owen) from the Kimmeridge Clay. *Palaeontology* **1**, 283–291 (1959).
24. Tarlo, L. B. A review of the Upper Jurassic pliosaurs. *Bull. Br. Museum (Natural Hist. Geol.* **4**, 145–189 (1960).
25. McHenry, C. R. 'Devourer of Gods'. The palaeoecology of the Cretaceous pliosaur *Kronosaurus queenslandicus*. Unpublished Ph.D. thesis, University of Newcastle. (University of Newcastle, 2009).
26. Holland, T. The mandible of *Kronosaurus queenslandicus* Longman, 1924 (Pliosauridae, Brachaucheniinae), from the Lower Cretaceous of Northwest Queensland, Australia. *J. Vertebr. Paleontol.* **e1511569**, 1–12 (2018).
27. Madzia, D., Sachs, S. & Lindgren, J. Morphological and phylogenetic aspects of the dentition of *Megacephalosaurus eulerti*, a pliosaurid from the Turonian of Kansas, USA, with remarks on the cranial anatomy of the taxon. *Geol. Mag.* 1–16 (2018) doi:10.1017/S0016756818000523.
28. Páramo-Fonseca, M. E., Gómez-Pérez, M., Noé, L. F. & Etayo-serna, F. *Stenorhynchosaurus munozi*, gen. et sp. nov. a new pliosaurid from the Upper Barremian (Lower Cretaceous) of Villa de Leiva, Colombia, South America. *Rev. la Acad. Colomb. Ciencias Exactas, Físicas y Nat.* **40**, 84–103 (2016).
29. Cau, A. & Fanti, F. A pliosaurid plesiosaurian from the Rosso Ammonitico Veronese Formation of Italy. *Acta Palaeontol. Pol.* **59**, 643–650 (2014).
30. Cau, A. & Fanti, F. High evolutionary rates and the origin of the Rosso Ammonitico Veronese Formation (Middle-Upper Jurassic of Italy) reptiles. *Hist. Biol.* 1–11 (2015) doi:10.1080/08912963.2015.1073726.
31. Fischer, V. *et al.* Peculiar macrophagous adaptations in a new Cretaceous pliosaurid. *R. Soc. Open Sci.* **2**, 150552 (2015).
32. Druckenmiller, P. S. Osteology of a new plesiosaur from the Lower Cretaceous (Albian) Thermopolis Shale of Montana. *J. Vertebr. Paleontol.* **22**, 29–42 (2002).
33. Albright, L. B., Gillette, D. D. & Titus, A. L. Plesiosaurs from the Upper Cretaceous (Cenomanian–Turonian) Tropic Shale of southern Utah, part 2: Polycotylidae. *J. Vertebr. Paleontol.* **27**, 41–58 (2007).
34. McKean, R. A new species of polycotylid plesiosaur (Reptilia: Sauropterygia)

from the Lower Turonian of Utah: Extending the stratigraphic range of *Dolichorhynchops*. *Cretac. Res.* **34**, 184–199 (2012).
35. Schumacher, B. A. A new polycotylid plesiosaur (Reptilia; Sauropterygia) from the Greenhorn Limestone (Upper Cretaceous; lower upper Cenomanian), Black Hills, South Dakota. *Geol. Soc. Am. Spec. Pap.* **427**, 133–146 (2007).
36. Schumacher, B. A. & Martin, J. E. *Polycotylus latipinnis* Cope (Plesiosauria, Polycotylidae), a nearly complete skeleton from the Niobrara Formation (Early Campanian) of southwestern South Dakota. *J. Vertebr. Paleontol.* e1031341 (2015) doi:10.1080/02724634.2015.1031341.
37. Bardet, N., Pereda Suberbiola, X. & Jalil, N.-E. A new polycotylid plesiosaur from the Late Cretaceous (Turonian) of Morocco. *Comptes rendus Palevol* **2**, 307–315 (2003).
38. O'Keefe, R. F. Cranial anatomy and taxonomy of Dolichorhynchops bonneri new combination, a polycotylid (Sauropterygia: Plesiosauria) from the Pierre Shale of Wyoming and South Dakota. *J. Vertebr. Paleontol.* **28**, 664–676 (2008).
39. Adams, D. A. *Trinacromerum bonneri*, new species, last and fastest pliosaur of the Western Interior Seaway. *Texas J. Sci.* **49**, 179–198 (1997).
40. Sato, T. A new polycotylid plesiosaur (Reptilia: Sauropterygia) from the Upper Cretaceous Bearpaw Formation in Saskatchewan, Canada. *J. Paleontol.* **79**, 969–980 (2005).
41. O'Gorman, J. P. & Gasparini, Z. Revision of *Sulcusuchus erraini* (Sauropterygia, Polycotylidae) from the Upper Cretaceous of Patagonia, Argentina. *Alcheringa An Australas. J. Palaeontol.* **37**, 163–176 (2013).
42. Frey, E. *et al.* A new polycotylid plesiosaur with extensive soft tissue preservation from the early Late Cretaceous of northeast Mexico. *Boletín la Soc. Geológica Mex.* **69**, 87–134 (2017).
43. Anderson, P. S. L., Friedman, M., Brazeau, M. D. & Rayfield, E. J. Initial radiation of jaws demonstrated stability despite faunal and environmental change. *Nature* **476**, 206–209 (2011).
44. Stubbs, T. L. & Benton, M. J. Ecomorphological diversifications of Mesozoic marine reptiles: the roles of ecological opportunity and extinction. *Paleobiology* 1–27 (2016) doi:10.1017/pab.2016.15.
45. MacLaren, J. A., Anderson, P. S. L., Barrett, P. M. & Rayfield, E. J. Herbivorous dinosaur jaw disparity and its relationship to extrinsic evolutionary drivers. *Paleobiology* **43**, 15–33 (2017).
46. Gans, C. The functional basis of the retroarticular process in some fossil reptiles. *J. Zool.* **150**, 273–277 (1966).
47. Taylor, M. Functional anatomy of the head of the large aquatic predator *Rhomaleaosaurus zetlandicus* (Plesiosauria, Reptilia) from the Toarcian (Lower Jurassic) of Yorkshire, England. *Philos. Trans. R. Soc. B Biol. Sci.* **335**, 247–280 (1992).
48. Dodson, P. Functional an ecological signficance of relative growth in Alligator. *J. Zool.* **175**, 315–355 (1975).
49. Busbey, A. B. The structural consequences of skull flattening in crocodilians. in *Functional morphology in vertebrate paleontology* (ed. Thomason, J. J.) 173–192 (Cambridge University Press, 1995).
50. Holzman, R. *et al.* Biomechanical trade-offs bias rates of evolution in the feeding apparatus of fishes. *Proc. R. Soc. B Biol. Sci.* **279**, 1287–1292 (2012).
51. Mccurry, M. R., Fitzgerald, E. M. G., Evans, A. R., Adams, J. W. & Mchenry, C.

R. Skull shape reflects prey size niche in toothed whales. *Biol. J. Linn. Soc.* **In Press**, 1–11 (2017).
52. Walmsley, C. W. *et al.* Why the Long Face? The Mechanics of Mandibular Symphysis Proportions in Crocodiles. *PLoS One* **8**, e53873 (2013).
53. Ridgway, S. H. & Harrison, R. *The Second Book of Dolphins and the Porpoise*. *Handbook of Marine Mammals* vol. 6 (Academic Press, 1999).
54. Massare, J. A. Tooth morphology and prey preference of Mesozoic marine reptiles. *J. Vertebr. Paleontol.* **7**, 121–137 (1987).
55. Foffa, D., Young, M. T., Stubbs, T. L., Dexter, K. G. & Brusatte, S. L. The long-term ecology and evolution of marine reptiles in a Jurassic seaway. *Nat. Ecol. Evol.* **2**, 1548–1555 (2018).
56. Zverkov, N. G., Fischer, V., Madzia, D. & Benson, R. B. J. Increased Pliosaurid Dental Disparity Across the Jurassic – Cretaceous Transition. *Palaeontology* **61**, 825–846 (2018).
57. Zeffer, A., Johansson, L. C. & Marmebro, Å. Functional correlation between habitat use and leg morphology in birds (Aves). *Biol. J. Linn. Soc.* **79**, 461–484 (2003).
58. Hinić-Frlog, S. & Motani, R. Relationship between osteology and aquatic locomotion in birds: determining modes of locomotion in extinct Ornithurae. *J. Evol. Biol.* **23**, 372–385 (2010).
59. Taylor, M. A. How tetrapods feed in the water: a funtional analysis by paradigm. *Zool. J. Linn. Soc.* **91**, 171–195 (1987).
60. Massare, J. A. Swimming capabilities of Mesozoic marine reptiles: implications for the methods of predation. *Palaeobiology* **14**, 187–205 (1988).
61. O'Keefe, F. R. A cladistic analysis and taxonomic revision of the Plesiosauria (Reptilia:Sauropterygia). *Acta Zool. Fenn.* **213**, 1–63 (2001).
62. Revelle, W. psych: Procedures for Psychological, Psychometric, and Personality Research. (2018).
63. Galili, T. dendextend: an R package for visualizing, adjusting, and comparing trees of hierarchical clustering. *Bioinformatics* **31**, 3718–3720 (2015).
64. R Core Team. R: A language and environment for statistical computing. (2016).
65. Wickham, H. *ggplot2: Elegant Graphics for Data Analysis*. (Springer-Verlag New York, 2016).
66. de Vries, A. & Ripley, B. D. ggdendro: Create Dendrograms and Tree Diagrams Using 'ggplot2'. (2016).
67. Slowikowski, K. ggrepel: Automatically Position Non-Overlapping Text Labels with 'ggplot2'. (2019).
68. Auguie, B. gridExtra: Miscellaneous Functions for 'Grid' Graphics. (2017).
69. Sievert, C. plotly for R. (2018).
